# A Randomized Trial of Lipid Metabolism Modulation with Fenofibrate for Acute Coronavirus Disease 2019

**DOI:** 10.21203/rs.3.rs-1933913/v1

**Published:** 2022-08-10

**Authors:** Julio Chirinos, Patricio Lopez-Jaramillo, Evangelos Giamarellos-Bourboulis, Gonzalo Dávila-del-Carpio, Abdul Bizri, Jaime Andrade-Villanueva, Oday Salman, Carlos Cure-Cure, Nelson Rosado-Santander, Mario Cornejo Giraldo, Luz González-Hernández, Rima Moghnieh, Rapti Angeliki, María Cruz Saldarriaga, Marcos Pariona, Carola Medina, Ioannis Dimitroulis, Charalambos Vlachopoulos, Corina Gutierrez, Juan Rodriguez-Mori, Edgar Gomez-Laiton, Rosa Pereyra, Jorge Ravelo Hernández, Hugo Arbañil, José Accini-Mendoza, Maritza Pérez-Mayorga, Haralampos Milionis, Garyfallia Poulakou, Gregorio Sánchez, Renzo Valdivia-Vega, Mirko Villavicencio-Carranza, Ricardo Ayala-Garcia, Carlos Castro-Callirgos, Rosa Alfaro Carrasco, Willy Lecca Danos, Tiffany Sharkoski, Katherine Greene, Bianca Pourmussa, Candy Greczylo, Jesse Chittams, Paraskevi Katsaounou, Zoi Alexiou, Styliani Sympardi, Nancy Sweitzer, Mary Putt, Jordana Cohen

**Affiliations:** University of Pennsylvania; Universidad de Santander; National and Kapodistrian University of Athens; Universidad Católica de Santa María; American University of Beirut; Hospital Civil de Guadalajara and Universidad de Guadalajara; Hospital of the University of Pennsylvania and Perelman School of Medicine, American University of Beirut; BIOMELAB S.A.S.; Hospital Nacional Carlos Alberto Seguín Escobedo; Hospital Carlos Alberto Seguín Escobedo; Hospital Civil de Guadalajara and Universidad de Guadalajara; Makassed General Hospital; SOTIRIA Athens General Hospital of Chest Disease; Hospital Nacional Adolfo Guevara Velasco; Hospital Nacional Edgardo Rebagliati Martins; Hospital Nacional Edgardo Rebagliati Martins; SOTIRIA Athens General Hospital of Chest Disease; National and Kapodistrian University of Athens; BIOMELAB S.A.S; Hospital Nacional Alberto Sabogal Sologuren; FOSCAL Internacional Floridablanca; Hospital Nacional Guillermo Almenara Irigoyen; Hospital Central Fuerza Aérea del Perú; Hospital Nacional Dos de Mayo; IPS Centro Científico Asistencial; Clínica de Marly; School Of Medicine, University Of Ioannina, Ioannina, Greece; National and Kapodistrian University of Athens; Fundación Cardiomet Cequin; Hospital Nacional Edgardo Rebagliati Martins; Hospital Nacional Edgardo Rebagliati Martins; HOSPITAL NACIONAL EDGARDO REBAGLIATI MARTINS; Hospital Nacional Alberto Sabogal Sologuren; Hospital Nacional Adolfo Guevara Velasco CUZCO; Hospital Nacional Adolfo Guevara Velasco CUZCO; Hospital of the University of Pennsylvania and Perelman School of Medicine; Hospital of the University of Pennsylvania and Perelman School of Medicine; Hospital of the University of Pennsylvania and Perelman School of Medicine; Hospital of the University of Pennsylvania and Perelman School of Medicine; University of Pennsylvania School of Nursing; National and Kapodistrian University of Athens; THRIASIO Eleusis General Hospital; THRIASIO Eleusis General Hospital; Washington University School of Medicine; Perelman School of Medicine. University of Pennsylvania.; University of Pennsylvania

**Keywords:** COVID-19, fenofibrate, fenofibric acid, lipid metabolism, SARS-CoV-2

## Abstract

**Background:**

Abnormal cellular lipid metabolism appears to underlie SARS-CoV-2 cytotoxicity and may involve inhibition of peroxisome proliferator activated receptor alpha (PPARα). Fenofibrate, a PPAR-α activator, modulates cellular lipid metabolism. Fenofibric acid has also been shown to affect the dimerization of angiotensin-converting enzyme 2, the cellular receptor for SARS-CoV-2. Fenofibrate and fenofibric acid have been shown to inhibit SARS-CoV-2 replication in cell culture systems *in vitro*.

**Methods:**

We randomly assigned 701 participants with COVID-19 within 14 days of symptom onset to 145 mg of fenofibrate (nanocrystal formulation with dose adjustment for renal function or dose-equivalent preparations of micronized fenofibrate or fenofibric acid) vs. placebo for 10 days, in a double-blinded fashion. The primary endpoint was a ranked severity score in which participants were ranked across hierarchical tiers incorporating time to death, duration of mechanical ventilation, oxygenation parameters, subsequent hospitalizations and symptom severity and duration. ClinicalTrials.gov registration: NCT04517396.

**Findings:**

Mean age of participants was 49 ± 16 years, 330 (47%) were female, mean BMI was 28 ± 6 kg/m^2^, and 102 (15%) had diabetes mellitus. A total of 41 deaths occurred. Compared with placebo, fenofibrate administration had no effect on the primary endpoint. The median (interquartile range [IQR]) rank in the placebo arm was 347 (172, 453) vs. 345 (175, 453) in the fenofibrate arm (P = 0.819). There was no difference in various secondary and exploratory endpoints, including all-cause death, across randomization arms. These results were highly consistent across pre-specified sensitivity and subgroup analyses.

**Conclusion:**

Among patients with COVID-19, fenofibrate has no significant effect on various clinically relevant outcomes.

## Introduction

Infection with severe acute respiratory syndrome coronavirus 2 (SARS-CoV-2), the virus responsible for coronavirus disease 2019 (COVID-19), is an important public health problem. Available data suggest that COVID-19 progression is dependent on metabolic mechanisms.^1^ Individuals infected with COVID-19 who developed acute respiratory distress syndrome (ARDS) and death are characterized by older age and a higher prevalence of hypertension, diabetes, and cardiovascular diseases compared to individuals with milder disease.^1–4^ Hyperglycemia and hyperlipidemia are also risk factors for acute respiratory distress in the setting of COVID-19.^1,5^ Indeed, type 2 diabetes mellitus and the metabolic syndrome are associated with a markedly increased risk of death in the setting of COVID-19.^1^

Several experimental studies suggest a mechanistic link between abnormal metabolism and the severity of SARS-CoV-2 and other coronavirus infections. Palmitoylation of the SARS-CoV-2 spike protein has been shown to be essential for viral-cell fusion and infectivity.^6–8^ Gene expression analyses in cultured human bronchial cells infected with SARS-CoV-2 as well as lung tissue from patients with COVID-19 indicated a marked shift in cellular metabolism, with excessive intracellular lipid generation.^9^ In further cell culture experiments, the PPARα-agonist fenofibrate (a widely available low-cost generic drug approved by the FDA and multiple other regulatory agencies around the world to treat dyslipidemias) reversed the metabolic changes induced by SARS-CoV-2, and inhibited viral production/replication.^9^ In more recent cell culture experiments, fenofibric acid, the active form of fenofibrate, induced destabilization of the SARS-CoV-2 viral spike (S) protein and reduction of viral infection.^10^ Fibrates also appear to exert immunomodulatory effects that could be beneficial in the setting of COVID-19.^11−13^

These pre-clinical studies suggesting that fenofibrate could directly target host metabolic pathways as well as viral proteins to minimize SARS-CoV-2 replication and possibly suppress its pathogenesis in respiratory tract tissue, motivated a rigorously-designed, international multi-center clinical trial to assess the potential efficacy of fenofibrate in COVID-19 in humans. The aim of this randomized controlled trial was to assess whether fenofibrate improves clinical outcomes in patients with COVID-19.

## Results

### Study Participants

**Figure 1:**
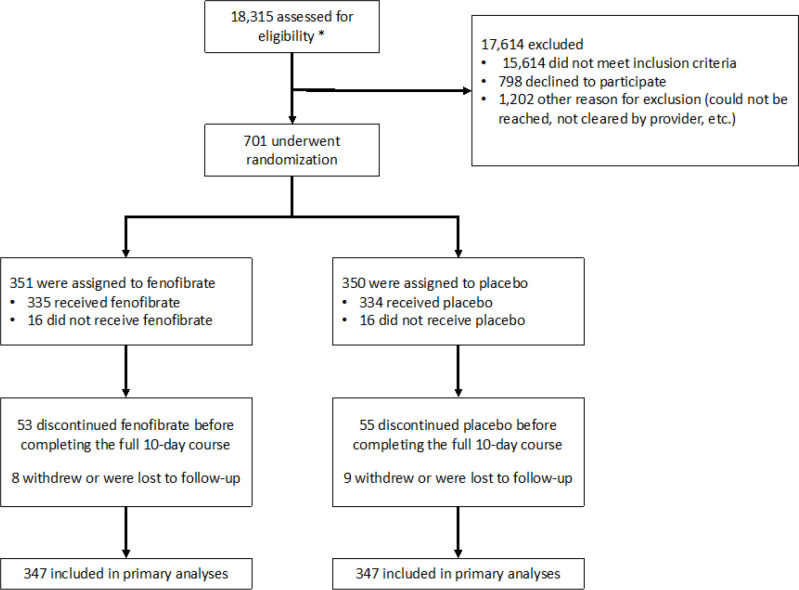
Participant Enrolment, Randomization, and Follow-up in the FERMIN trial * Pre-screening information was only available for the Penn site due to regulatory limitations and lack of collection at other sites

A total of 701 participants were enrolled and randomized (156 in Colombia, 133 in Greece, 116 in the USA, 116 in Peru, 113 in Lebanon, and 67 in Mexico). General characteristics of study participants are shown in Table 1. The mean age of enrolled participants was 49±16 years, 330 (47%) were female, mean BMI was 28±6 kg/m^2^, 102 (15%) had a history of diabetes mellitus, 47 (7%) had a history of ischemic heart disease, 186 (27%) participants had a history of hypertension, and 302 (43%) were enrolled as inpatients. A total of 351 participants were randomized to fenofibrate and 350 participants were randomized to placebo (Figure 1). Only 17 participants (2%) were excluded, withdrew following randomization or were lost to follow-up. Most participants (n=438; 62%) participants were positive for SARS-CoV-2 by real time polymerase chain reaction testing and the remaining were positive by rapid antigen testing.

### Primary Endpoint

**Figure 2:**
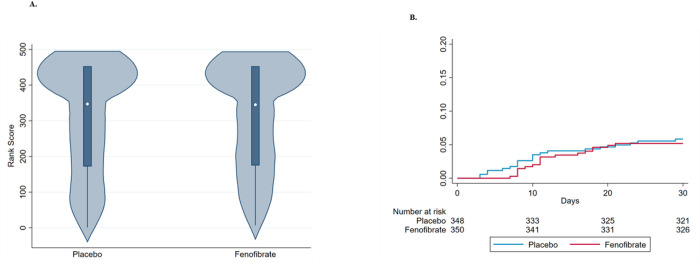
Key Outcomes Among Participants in Each Randomization Arm **Panel A** shows the distribution of the primary endpoint (ranked severity score) between the randomization arms. The y-axis represents the range of ranked severity scores, and the x-axis represents the frequency density of distributions of the ranks in each treatment arm. The white dot represents the median ranked severity score, the solid box represents the interquartile range, and the vertical lines represent the upper- and lower-adjacent values. The upper adjacent value and the interquartile range values were identical. **Panel B** shows the cumulative incidence for all-cause death at 30-days.

In the primary intent-to-treat analyses, the distribution of the ranked severity scores between participants assigned to fenofibrate vs. placebo was remarkably similar. The median (interquartile range [IQR]) ranked severity score in the placebo arm was 347 (172, 453) vs. 345 (175, 453) in the fenofibrate arm (P=0.819), where a lower value signifies more severe COVID-19 course (Table 2 and Figure 2A). After adjusting for age, sex, inpatient vs. outpatient status, baseline FiO_2_/SpO_2_, race, ethnicity, BMI, baseline diabetes status, and country, and clustered by site, subjects assigned to fenofibrate exhibited mean ranked severity scores that were 0.09 (95% CI −0.04, 0.21) units higher than those assigned to control (P-value=0.152). The individual components of the ranked severity score are described in Table S1.

### Secondary and Exploratory Endpoints

The number of days alive, out of the intensive care unit, free of mechanical ventilation (invasive and non-invasive), extracorporeal membrane oxygenation (ECMO) or maximal available respiratory support in the 30 days following randomization was similar among the arms (median time in both arms, 30 [IQR 30, 30]; P=0.134). The seven-category WHO ordinal scale was similar between the arms (placebo median 1 [IQR 1, 2]; fenofibrate median 1 [IQR 1, 1]; P-value 0.246). Similarly, the modified ranked severity scores (constructed like the primary endpoint but using a more comprehensive COVID-19 symptom scale instead of the dyspnea Borg scale) were very similar across arms (placebo median score 358 [IQR 174, 513]; fenofibrate median score 343 [IQR 177, 525]; P-value 0.740).

Kaplan-Meier curves for deaths in the two arms are shown in Figure 2B. A total of 41 deaths occurred; 22 in the placebo arm and 19 in the fenofibrate arm (hazard ratio, 0.880 [95%CI=0.465, 1.663]; P-value=0.693). After adjusting for age, sex, inpatient vs. outpatient status, baseline FiO_2_/SpO_2_, race, ethnicity, BMI, baseline diabetes status, and country, and clustered by site, there was no significant difference in all-cause death at 30 days between the arms (adjusted HR 0.983; 95% CI 0.562, 1.718; P-value 0.952). The Kaplan-Meier failure estimates were not significantly different between the arms (P-value=0.692).

The number of days alive and out of the hospital during the 30 days following randomization were similar between the two arms (median days in the placebo arm, 30 [IQR 25, 30]; median days in the fenofibrate arm, 30 [IQR 25, 30]; P-value=0.834). The additional modified ranked severity score (similar to the primary endpoint, but built only with factors 1–4) was similar across the arms (placebo median score 317 [IQR 172, 317]; fenofibrate median score 317 [IQR 175, 317]; P-value=0.836). In analyses restricted to the 398 participants enrolled as outpatients, the risk of hospitalization was not significantly different in participants randomized to fenofibrate compared with placebo (1 vs. 4 participants hospitalized; unadjusted HR 0.249; 95% CI 0.028, 2.227; P-value 0.214; Figure S1). In analyses restricted to the 302 participants enrolled as inpatients, the cause-specific hazard for hospital discharge, considering death as a competing risk, was essentially identical between the arms (unadjusted HR 1.001; 95% CI 0.792, 1.267; P-value=0.990; Figure S2).

Analyses of all secondary and exploratory endpoints via pre-specified linear regression analyses that adjusted for age, sex, inpatient vs. outpatient status, baseline FiO_2_/SpO_2_, race, ethnicity, BMI, baseline diabetes status, and country and clustered by site, were consistent with the primary results obtained with the van Elteren test (Table 2).

### Pre-specified Subgroup Analyses

**Figure 3:**
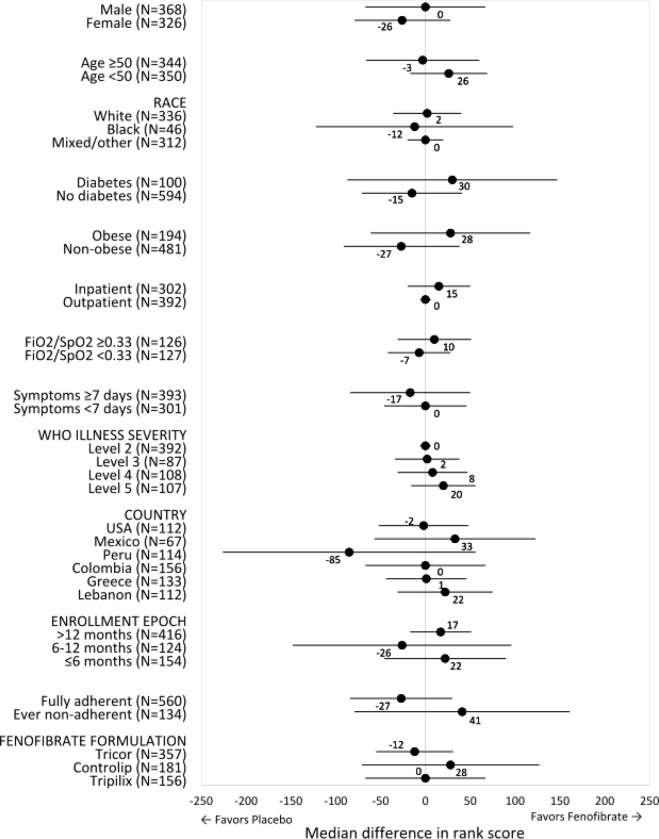
Forest Plot of the Differences in Ranked Severity Scores Across Subgroups The dots represent the differences in median ranked severity scores between participants randomized to fenofibrate vs. placebo in each subgroup. Positive values indicate better outcomes in the fenofibrate arm. The bars represent the 95% confidence intervals.

As shown in detail in Figure 3, there was no evidence of effect modification by age, sex, race, diabetes status, obesity status, inpatient vs. outpatient status at the time of enrolment, FiO_2_/SpO_2_ at the time of enrollment (< vs. ≥ median), duration of symptoms (<7 vs. ≥7 days), WHO disease severity, country, formulation, adherence to therapy, or compound (fenofibrate vs. fenofibric acid).

### Adverse Events

There were 61 (17%) adverse events in the placebo arm compared with 46 (13%) in the fenofibrate arm. Adverse events are summarized in Table S2. There were no appreciable differences in the incidence of adverse events classified by organ system, except for a slightly greater incidence of gastrointestinal adverse events with fenofibrate (9 events [3%] in the placebo arm; 19 [5%] in the fenofibrate arm).

## Discussion

We performed an international multicenter randomized placebo-controlled clinical trial designed to evaluate the clinical efficacy of fenofibrate on COVID-19 severity. Our trial, which enrolled both inpatients and outpatients, did not demonstrate any appreciable effect of fenofibrate on the trial primary endpoint, which evaluated multiple facets of COVID-19 severity, including death, invasive and non-invasive mechanical ventilatory support, duration of hospitalization among inpatients, time to hospitalization among outpatients, and symptom severity among outpatients who were not hospitalized. Multiple prespecified sensitivity, secondary, and subgroup analyses corroborated the primary analyses. Over 30 days of follow-up after randomization, we observed no significant effect of fenofibrate therapy on the number of days alive, out of the ICU, and free of invasive mechanical ventilation; on the WHO ordinal outcome scale; nor on number of days alive and out of the hospital. Similarly, there was no significant difference observed when the ranked severity score incorporated a multifactorial COVID-19 symptom score instead of the Borg score or if the ranked severity score was restricted to only objective outcome measures (i.e., when the symptom score was omitted).

Our study was motivated by various epidemiologic and *in vitro* observations suggesting a link between abnormal lipid metabolism and the pathogenesis of SARS-CoV-2 infection or severity of COVID-19, as well as *in vitro* studies in which an antiviral effect of fenofibrate has been reported. Abnormal lipid metabolism has been shown to be involved in the cellular pathogenesis SARS-CoV-2 and other RNA viruses. Nardacci *et al* demonstrated that SARS-CoV-2 infection induces a striking accumulation of lipid droplets in cultured Vero E6 cells, as well as type II pneumocytes from infected patients.^14^ Although this phenomenon was reported to constitute a major difference compared to SARS-CoV-1 infection, it does not appear to be unique to SARS-CoV-2, since marked alterations in lipid metabolism have been shown to occur as a consequence of HCV infection,^15^ as well as human coronavirus 229E (HCoV-229E)^16^ and some picornaviruses.^17^

Given the potential role of dysregulated lipid metabolism in SARS-CoV-2 infection, there is significant interest in the potential antiviral role of medications that affect lipid metabolism. Davies *et al* recently reported an effect of fenofibric acid (the active metabolite of fenofibrate) on the dimerization of angiotensin-converting enzyme 2 (ACE2), the cellular receptor for SARS-CoV-2.^10^ Fenofibric acid was also reported to destabilize the receptor-binding domain (RBD) of the SARS-CoV-2 spike protein and to inhibit binding of the S protein RBD to ACE2. The investigators subsequently assessed the effect of fenofibrate and fenofibric acid in cultured Vero cells infected with SARS-CoV-2. They reported that both fenofibrate and fenofibric acid were able to reduce viral infection rates. However, the relative effect of fenofibrate vs. fenofibric acid appeared to vary across experimental assays, which also utilized one of 2 different SARS-CoV isolates. A preliminary non-peer reviewed publication by Elrich *et al* ^9^ reported the results of gene expression analyses in cultured human bronchial cells infected with SARS-CoV-2 and lung tissue from patients with COVID-19, demonstrating a marked shift in cellular metabolism and excessive intracellular lipid generation in infected cells. In this report, the transcriptional response to SARS-CoV-2 involved predominantly metabolic genes and was characterized by changes in pathways of endoplasmic reticulum stress, upregulation of glycolysis and dysregulation of the citric acid cycle, upregulation of fatty acid and cholesterol synthesis, and the suppression of fatty acid oxidation. In further cell culture experiments, fenofibrate was reported to reverse the metabolic changes induced by SARS-CoV-2, and inhibited viral production/replication.^9^ Interestingly, despite the potential impact of PPAR-α activation on cell metabolism in infected cells, Davies *et al* reported that the PPAR-α antagonist GW6471 did not appreciably alter the antiviral actions of fenofibrate in one of their cell culture systems,^10^ suggesting that the antiviral activity of fenofibrate measured in their assays was not mediated by this transcription factor. In addition to its potential antiviral activity, fenofibrate may exert immunomodulatory effects that could have an impact in COVID-19.^11–13^

Despite the potential effects of fenofibrate on SARS-CoV-2 reported using *in vitro* culture systems, our randomized trial convincingly demonstrates the absence of an appreciable clinical benefit on all endpoints studied. There was near complete overlap between the 2 trial arms in our primary endpoint, which incorporates multiple clinically relevant aspects of COVID-19 severity, including death, invasive and non-invasive mechanical ventilatory support, duration of hospitalization among inpatients, time to hospitalization among outpatients, and symptom severity among outpatients who were not hospitalized. Similarly, there was no benefit of fenofibrate on multiple prespecified secondary and exploratory endpoints, as well as in sensitivity analyses and pre-specified subgroup analyses. Importantly, the findings were consistent across inpatients and outpatients, across countries, among patients who initiated treatment < 7 days from the onset of symptoms and those who initiated therapy at a later time, across strata of body mass index, in diabetic and non-diabetic participants, in males and females, across age strata, race, or according to the specific formulation utilized. The clear lack of a clinical benefit in our double blinded randomized trial contrasts with the various *in vitro* effects reported as detailed above. Importantly, the pathogenesis of COVID-19 is complex and involves not only primary cytotoxic effects of SARS-CoV-2 but also a complex set of systemic host responses that involve the innate and acquired immune systems, various neurohormonal canonical pathways, as well as multiorgan damage and failure.^18–20^ Therefore, *in vitro* cellular effects of drugs may fail to translate into clinical benefit as a result of a wide host of potential pathophysiological phenomena in whole organisms. Our trial reinforces the importance of not equating *in vitro* efficacy against SARS-CoV-2 with clinical efficacy in the setting of COVID-19, and further demonstrates the importance of performing rigorous prospective randomized controlled trials to assess the potential clinical benefit of interventions for COVID-19 prior to clinical implementation.

Our study also provides important safety data for this widely available medication. Although a trend towards a higher incidence of gastrointestinal side effects was observed, our trial demonstrates that fenofibrate therapy was not associated with an excess of major adverse events. These findings suggest that this medication can be safely administered or continued in patients with COVID-19 who require it for other indications, such as dyslipidemia.

Our study is strengthened by the use of a double-blinded, placebo-controlled, randomized study design which overcomes the problem of confounding due to multiple known or unknown uncontrolled factors, as occurs in observational studies. We enrolled participants across multiple international centers with diverse, global representation of individuals affected by COVID-19. Participants were recruited as both inpatients and outpatients from medical centers in diverse settings, using a pragmatic approach to capture data collected during routine care, further contributing to generalizability of the results to a broad population of those susceptible to COVID-19. There were low rates of attrition of study participants and high rates of adherence to the study medication, which were similar across the randomization arms. The use of the ranked severity score as the primary endpoint incorporated several clinical events highly relevant to patients with COVID-19 into a single outcome measure. Our relatively large sample size further facilitated evaluation for clinically significant differences in several secondary endpoints. There are also notable limitations. The study enrolled participants over an 18-month period during which there were several different dominant COVID-19 variants and management strategies as well as introduction of vaccines, all of which varied across countries at different timepoints. We attempted to address this by adjusting for country and epoch (i.e., time since trial initiation) and clustering by study site in secondary analyses to account for differences in treatment practices over time and by location. These adjustments did not significantly impact our findings. We also performed subgroup analyses which demonstrated no meaningful differences across locations and epochs.

In conclusion, in our multicenter randomized placebo-controlled trial, fenofibrate did not exert any appreciable clinical benefits among patients with COVID-19. Further studies are required to assess whether various other interventions designed to affect cellular metabolic pathways can impact clinical outcomes in COVID-19.

## Methods

### Study Design and Oversight

The FEnofibRate as a Metabolic INtervention for COVID-19 (FERMIN) trial was a prospective multicenter randomized double-blinded trial conducted at 25 centers in 6 countries (United States, Mexico, Greece, Peru, Colombia, and Lebanon). Participants were enrolled from October 2, 2020 to February 27, 2022. A data coordinating center at the University of Pennsylvania oversaw data management and statistical analyses. The trial design was approved by the ethics committee of each participating center, or in the US, via reliance agreements with a central institutional review board (University of Pennsylvania). An independent data safety monitoring board was also assembled to provide oversight of the trial. All participants provided written or electronic informed consent. The trial was registered at ClinicalTrials.gov (registration: NCT04517396).

### Participants

Participants being evaluated in emergency departments, outpatient clinics, other urgent/emergent care settings, or admitted to the hospital with COVID-19, were assessed for eligibility.

Participants were required to: (1) be a minimum of 18 years of age; (2) carry a diagnosis of COVID-19, based on: (a) a compatible clinical presentation with a positive laboratory test for SARS-CoV-2, or (b) considered by the primary team to be a Person Under Investigation undergoing testing for COVID-19 with a high clinical probability, in addition to compatible pulmonary infiltrates on chest x-ray (bilateral, interstitial or ground glass opacities) or chest CT; (3) have fewer than 14 days since symptom onset; (4) be able to provide informed consent.

Exclusion criteria were as follows: (1) known pregnancy or breastfeeding; (2) estimated glomerular filtration rate (eGFR) < 30 mL/min/1.73 m^2^ or undergoing dialysis (chronic kidney disease stages 4–5); given the lack of available preparations for participants with eGFR < 60 mL/min/1.73 m^2^ in trial countries other than the USA (see supplemental section), the latter eGFR cut-point was implemented for exclusion in those countries; (3) history of active liver disease, cholelithiasis, uncontrolled hypothyroidism, or rhabdomyolysis (suspected or confirmed); (4) known hypersensitivity to fenofibrate or fenofibric acid; (5) ongoing treatment with fenofibrate, clofibrate, warfarin and other coumarin anticoagulants, glimepiride, cyclosporine, tacrolimus; (6) use of statins other than simvastatin, pravastatin or atorvastatin ≤ 40 mg/d or rosuvastatin ≤ 20 mg/d; (7) prisoners/incarcerated individuals; (8) inability to read, write or no access to a smart phone, computer or tablet device (at sites where eConsenting was performed to minimize risk of COVID-19 exposure to study staff); (9) intubated patients.

### Randomization, Blinding, and Treatment Allocation

See Figure S3 for the general workflow of the trial. Eligible participants were randomized 1:1 to either: (1) fenofibrate (or its active metabolite, fenofibric acid) administered for 10 days, ^21,22^ with appropriate dose reductions or exclusions implemented for patients with chronic kidney disease as per the approved preparation label; or (2) placebo of similar appearance. These interventions were added to usual care. Participants and investigators were blinded to the randomized intervention. Treatment allocation was concealed using a secure web-based randomization system. Permuted block randomization was performed in randomly varying block sizes by clinical site, sex, age group (< 65 or ≥ 65 years) and inpatient vs. outpatient status. All investigators collecting information on clinical endpoints were blinded to the study intervention. The specific drug preparations used in various countries are detailed in the Online Supplement.

Criteria for study medication discontinuation included any of the following occurring at any time during the administration period: (1) acute kidney injury with eGFR < 30 ml/m^2^/min; (2) suspected or confirmed rhabdomyolysis; (3) red or brown urine, which may indicate myoglobinuria, unless considered by the investigator to be clearly not due to rhabdomyolysis (for instance, in the presence of normal circulating creatine kinase); (4) liver failure or increased AST or ALT to > 3 times the upper limit of normal. In cases in which the medication was discontinued, data collection continued normally as per the usual study protocol.

### Follow-up and outcomes

The ‘on study’ period was 30 days. For participants randomized as inpatients, daily assessments (via medical record review) were performed to assess clinical status, with particular attention to the study endpoints (death, mechanical ventilation and the FiO_2/_SpO_2_ ratio), until hospital discharge or 30 days (whichever was shortest). For participants discharged prior to 30 days post-randomization, a follow-up call at the ~ 30-day time point assessed vital and functional status, symptoms and major adverse events, including hospitalizations. For participants randomized as outpatients or discharged within 24 hours of receiving the first dose of the study medication, participants were called at ~ 5-, 10-, 15- and 30-days post-randomization, in order to assess vital and functional status, hospitalization status, the severity of dyspnea (via the modified Dyspnea Borg Scale) and major adverse events.

### Primary endpoint

**Figure 4:**
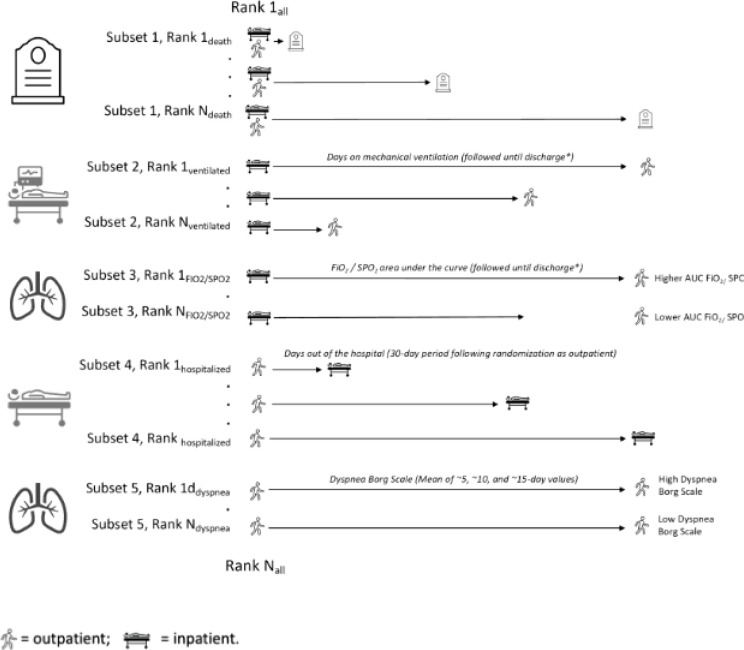
Primary endpoint of the trial (ranked severity score)

The primary endpoint of the trial (Fig. 4) was a global severity score that hierarchically ranked participant outcomes according to 5 factors: (1) time to death (ranked from shortest to longest, up to 30 days post-randomization), (2) the number of days supported by mechanical ventilation (invasive or non-invasive) or extracorporeal membrane oxygenation (until hospital discharge, up to 30 days post-randomization, ranked from longest to shortest); (3) the inspired concentration of oxygen/percent oxygen saturation (FiO_2/_SpO_2_) ratio area under the curve (until hospital discharge, up to 30 days post-randomization, ranked from highest to lowest); (4) for participants enrolled as outpatients who were subsequently hospitalized, the number of days out of the hospital during the 30 day-period following randomization (ranked from lowest to highest); (5) for participants enrolled as outpatients who did not get hospitalized during the 30-day observation period, the modified Borg dyspnea scale (mean value of assessments at ~ 5, ~10 and ~ 15 days). Patients who are enrolled as inpatients and discharged within 24 hours of receiving the first dose of the study medication were ranked similarly to outpatients.

The ranked severity score has several advantages compared to binary outcomes (e.g., all-cause death) or time-to-event outcomes (e.g., time to death).^23–25^ It incorporates information about each of the highest-priority events in COVID-19, but allows these events to be prioritized within a single endpoint. For instance, the principal outcome of interest is death, but even if there is no difference in rate of death, we would still be interested in a shorter duration of invasive respiratory support, a shorter duration of hospital admission with better oxygenation parameters, and so on. This maximizes study power and minimizes the number of participants that need to be enrolled in order to detect clinically-meaningful differences.

### Secondary and exploratory endpoints

Secondary endpoints were as follows: (1) the number of days alive, out of the intensive care unit, free of mechanical ventilation (invasive and non-invasive), extracorporeal membrane oxygenation (ECMO) or maximal available respiratory support in the 30 days following randomization; (2) a seven-category ordinal scale consisting of the following categories: not hospitalized with resumption of normal activities; not hospitalized, but unable to resume normal activities; hospitalized, not requiring supplemental oxygen; hospitalized, requiring supplemental oxygen; hospitalized, requiring nasal high-flow oxygen therapy, noninvasive mechanical ventilation, or both; hospitalized, requiring extracorporeal membrane oxygenation (ECMO), invasive mechanical ventilation, or both; and, death; (3) a ranked severity score similar to the primary endpoint, but using a more comprehensive COVID-19 symptom scale instead of the dyspnea Borg scale (Appendix 1).

Exploratory endpoints included: (1) time to all-cause death; (2) time to hospitalization (among participants enrolled as outpatients); (3) time to discharge (among participants enrolled as inpatients); (4) the number of days alive and out of the hospital during the 30 days following randomization; (5) a ranked severity score similar to the primary endpoint, but built only with factors 1–4.

### Statistical analyses

Analyses were performed on an intention-to-treat basis. The primary analyses used the non-parametric two-sided Wilcoxon rank sum test to compare the distribution of severity scores across treatment arms. In a prespecified, secondary analysis of the primary endpoint we used linear regression to compare mean ranked severity scores between arms after adjustment for age, sex, inpatient vs. outpatient status at enrollment, FiO_2/_SpO_2_ at the time of enrollment, race, ethnicity, body mass index (BMI), history of diabetes at baseline, and country (to account for differences in treatment practices, timing of variants, and timing of surges), with cluster robust standard errors to account for clustering by site. We evaluated time-to-event outcomes using Cox proportional hazards models from the time of enrollment and censored at the end of the 30-day follow-up period. We assessed for violation of the proportional hazards assumption using Schoenfeld residuals and planned to incorporate a time-by-treatment interaction term if the assumption was violated. In analyses that did not include death as part of the time-to-event outcome, we evaluated cause-specific hazards to address death as a competing risk.

We performed prespecified exploratory subgroup analyses according to sex, age (categorized by < or ≥ the median value in the study population), race, presence of pre-existing diabetes, BMI (categorized by obese or non-obese), inpatient vs. outpatient status at the time of enrollment, FiO2/SpO2 at the time of enrollment (categorized by < or ≥ the median value in the study population), duration of symptoms prior to randomization (< 7 vs. ≥7 days), country, baseline COVID-19 severity based on the World Health Organization (WHO) criteria,^28^ and fenofibrate formulation, using the two-sided van Elteren test to compare severity scores stratified by these prespecified subgroups.^26,27^

The analysis was based on complete cases, and ignored missing data. This approach was prespecified based on a missingness rate of less than 5% (see Supplemental Methods). The two-sided type I error rate was 0.05 and was not adjusted for multiple comparisons except for the primary analysis, which included an interim analysis; confidence intervals (CI) were at the 95% level. Analyses were performed using Stata version 16.1 (StataCorp, College Station, TX).

### Power calculation

Using Monte Carlo simulations to apply likely distributions of participants across each of the hierarchies based on available published data,^2,29,30^ we estimated that the trial would have 80% power at an alpha of 0.0492 (allowing for one interim analysis at 50% of enrollment with an alpha of 0.0054^31,32^) to demonstrate an 11% difference in median ranked severity scores between the treatment arms at the target sample size of 700. Power calculations were performed using Python and PASS16.^33^ Power calculations for other endpoints are presented in the supplemental section.

### Role of the Funding Source

This trial was sponsored by the National Center for Advancing Translational Sciences through a cooperative agreement (U01) grant to the University of Pennsylvania, and by a grant provided by Abbott Product Operations AG (Allschwil, Switzerland). The industry sponsor had no role in study design, clinical data collection, outcome adjudication or data analysis.

## Supplementary Material

Supplement 1

## Figures and Tables

**Table 1.** General Characteristics of Study participants

Variable		Total (n=701)	Placebo (n=350)	Fenofibrate (n=351)
Age, years	Mean (SD)	49 (16)	49 (16)	49 (16)
Female sex		330 (47%)	164 (47%)	166 (47%)
Race/Ethnicity	Non-Hispanic Black	47 (7%)	23 (7%)	24 (7%)
	Non-Hispanic White	263 (38%)	135 (39%)	128 (37%)
	Hispanic	350 (50%)	173 (49%)	177 (51%)
	Other	40 (6%)	19 (5%)	21 (6%)
Hypertension		186 (27%)	98 (28%)	88 (25%)
Diabetes mellitus		102 (15%)	58 (17%)	44 (13%)
On insulin		20 (20%)	12 (21%)	8 (18%)
High cholesterol		96 (14%)	56 (16 %)	40 (11%)
Ischemic heart disease		47 (7%)	31 (9%)	16 (5%)
Heart failure		19 (3%)	9 (3%)	10 (3%)
Atrial fibrillation		14 (2%)	8 (2%)	6 (2%)
Prior pulmonary embolism or deep vein thrombosis		14 (2%)	9 (3%)	5 (1%)
Obstructive sleep apnea		23 (3%)	15 (4%)	8 (2%)
Chronic pulmonary disease		82 (12%)	43 (12%)	39 (11%)
Current smoker		67 (10%)	34 (10%)	33 (9%)
				6 (2%)
Illicit drug use		11 (2%)	5 (1%)	
Dyspnea severity, 0–10 Borg scale	Mean (SD)	2.2 (2.6)	2.3 (2.7)	2.0 (2.5)
Cough severity, 0–10 scale	Mean (SD)	3.8 (2.9)	3.8 (3.0)	3.8 (2.8)
Chest pain severity, 0–10 scale	Mean (SD)	1.6 (2.5)	1.7 (2.6)	1.5 (2.4)
Myalgias severity, 0–10 scale	Mean (SD)	2.9 (3.0)	3.0 (3.1)	2.8 (2.9)
Fatigue severity, 0–10 scale	Mean (SD)	3.6 (3.1)	3.6 (3.0)	3.6 (3.2)
Multifocal infiltrates on chest imaging		185 (62%)	88 (62%)	97 (61%)
Oxygen saturation, %	Mean (SD)	96 (3)	96 (3)	96 (3)
Systolic blood pressure, mmHg	Mean (SD)	123 (16)	123 (16)	123 (16)
Diastolic blood pressure, mmHg	Mean (SD)	76 (31)	76 (31)	76 (30)
Heart rate, beats per minute	Mean (SD)	82 (13)	82 (14)	82 (12)
BMI, kg/m^2^	Mean (SD)	28 (6)	28 (6)	28 (6)
eGFR, mL/min/1.73m^2^	Mean (SD)	101 (20)	101 (21)	100 (20)
Leukocyte count, 10^9^ cells/L	Mean (SD)	1287 (3053)	1346 (3147)	1231 (2965)
Platelets, 10^3^ cells/μL	Mean (SD)	227 (86)	226 (91)	228 (81)
C-reactive protein, mg/dL	Mean (SD)	43 (73)	42 (68)	45 (78)
Days from admission to randomization	Mean (SD)	2 (3)	2 (3)	2 (2)
Days from symptom onset to ran	Mean (SD)	7 (4)	7 (4)	7 (4)
Inpatient vs Outpatient	Inpatient	302 (43%)	151 (43%)	151 (43%)
	Outpatient	398 (57%)	199 (57%)	199 (57%)
Total days on drug	Mean (SD)	9 (3)	9 (3)	9 (3)
Dropouts		17 (2%)	9 (3%)	8 (2%)

*Abbreviations:* BMI=body mass index; eGFR=estimated glomerular filtration rate; SD=standard deviation

**Table 2.** Comparison of secondary and exploratory endpoints between fenofibrate vs. placebo* Linear regression models were prespecified and adjusted for age, sex, inpatient vs. outpatient status, baseline FiO2/SpO2, race, ethnicity, BMI, baseline diabetes status, and country, and clustered by site.

Endpoint	Wilcoxon rank sum test			Linear regression	
	Fenofibrate Median (IQR)	Placebo Median (IQR)	P value	Effect size β (95% CI)	P value
**Secondary Endpoints**					
Number of days alive, out of ICU/ECMO/invasive ventilation	30 (30, 30)	30 (30, 30)	0.134	1.23 (−1.54, 4.00)	0.361
WHO ordinal scale	1 (1, 1)	1 (1, 2)	0.246	−0.149 (−0.36, 0.06)	0.156
Modified ranked severity score (COVID-19 symptom scale instead of Borg)	5.05 (2.98. 5.22)	5.05 (2.98, 5.21)	0.928	0.083 (−0.04, 0.20)	0.168
**Exploratory Endpoints**					
Number of days alive and out of the hospital at 30 days	30 (25, 30)	30 (25, 30)	0.834	0.29 (−1.84, 2.41)	0.779
Modified ranked severity score with only factors 1–4	5.03 (2.98, 5.03)	5.03 (2.98, 5.03)	0.776	0.082 (−0.04, 0.20)	0.169

* Linear regression models were prespecified and adjusted for age, sex, inpatient vs. outpatient status, baseline FiO2/SpO2, race, ethnicity, BMI, baseline diabetes status, and country, and clustered by site.

For analyses related to all cause-death, hospitalization, and discharge, refer to the main text (Results section)

*Abbreviations*: CI=confidence interval; ECMO=extracorporeal membrane oxygenation; ICU=intensive care unit; IQR=interquartile range; WHO=World Health Organization

## Data Availability

The trial data is not publicly available but may be made available for scientific collaborations after the execution of appropriate data sharing agreements.
