# Supplementary material for: A Randomized Trial of Lipid Metabolism Modulation with Fenofibrate for Acute Coronavirus Disease 2019
Source: Res Sq. 2022 Aug 10:rs.3.rs-1933913. Preprint. [Version 1] doi: 10.21203/rs.3.rs-1933913/v1 (PMC9387540; doi:10.21203/rs.3.rs-1933913/v1)
Supplement: Supplement 1 [file FERMINOnlineSupplementFINALclean.docx]

**A Randomized Trial of Lipid Metabolism Modulation with Fenofibrate for Acute Coronavirus Disease 2019**

**Supplemental Materials**

**Contents**

[Supplemental Methods 2](#_Toc110586790)

[Power calculations 2](#_Toc110586791)

[Additional Details of the Analytic Plan 2](#_Toc110586792)

[*Primary Analyses* 2](#_Toc110586793)

[*Per-Protocol Analyses* 3](#_Toc110586794)

[*Secondary Analyses* 3](#_Toc110586795)

[*Subgroup Analyses* 3](#_Toc110586796)

[*Missing Data* 3](#_Toc110586797)

[Adverse Events 4](#_Toc110586798)

[*Adverse Event Reporting* 4](#_Toc110586799)

[*Key definitions* 4](#_Toc110586800)

[*Classification of AEs* 4](#_Toc110586801)

[Protocol Deviations 5](#_Toc110586802)

[Study Drug Preparations 6](#_Toc110586803)

[Table S1. Components of the global rank score by study arm 7](#_Toc110586804)

[Table S2. Adverse events by study arm 8](#_Toc110586805)

[Figure S1. Kaplan Meier curve of time to hospitalization (among participants enrolled as outpatients) 9](#_Toc110586806)

[Figure S2. Cumulative incidence curve of time to discharge (among participants enrolled as inpatients) 10](#_Toc110586807)

[Figure S3. General workflow of the trial 11](#_Toc110586808)

[Supplemental References 12](#_Toc110586809)

## Supplemental Methods

## Power calculations

We enrolled 700 participants. Participants were randomized to one of 2 interventions (fenofibrate plus usual care vs. placebo plus usual care). Assuming feasible distributions of participants across each of the five hierarchies from the available published evidence,^1-3^ we performed 10,000 simulations of rank distributions of 700 participants with varying distributions of endpoint parameters, and determined that there would be 80% power at an alpha of 0.0492 (allowing for one interim analysis at 50% of enrollment with an alpha of 0.0054^4,5^) to observe an 11% difference in median rank scores between the treatment arms. Power calculations were performed using Python and PASS16.^6^ Statistical code for the power calculations can be obtained by contacting the data coordinating center (jco@pennmedicine.upenn.edu).

Enrollment of 700 participants increased our power for secondary outcomes. For the secondary outcome of number of days alive, out of the intensive care unit, free of mechanical ventilation (invasive and non-invasive), extracorporeal membrane oxygenation (ECMO) or maximal available respiratory support in the 30 days following randomization, assuming a standard deviation of 7 (the SD from the REPLACE COVID trial for this outcome),^7^ a sample size of 700 would have 90% power to detect mean difference of 1.7 days for fenofibrate versus placebo.^8,9^ Assuming that the SD may be higher in the current study with the inclusion of outpatients, we estimated that we would have sufficient power to estimate a clinically significant 3.4 day mean difference between treatment arms with a SD of 14.

For the secondary outcome of the WHO ordinal scale, based on the ordinal scale distributions from the Remdesivir trial,^10^ we estimated that we would have 90% power to observe an OR of 1.5 for clinical improvement in the ordinal scale.^8,11^ Given the expected lower severity of illness in our participants, with an adjusted reasonable distribution towards the lower end of the scale, we would have 90% power to observe an OR of 1.7.

For the exploratory outcome of all-cause death, assuming a death rate of 14% (observed in REPLACE COVID),^7^ we estimated that we would have 80% statistical power to detect a HR of 1.4 or larger.^12,13^ Considering the mixed inclusion of inpatients and outpatients, if the death rate is 5%, we would have sufficient statistical power to detect a HR of 1.7 or larger.

For the exploratory outcome of number of days alive and out of the hospital, assuming a standard deviation of 8 (the SD from the REPLACE COVID trial for this unpublished outcome),^7^ we estimated that a sample size of 700 would have 90% power to determine a mean difference of at least two days between treatment arms.^8,9^ Assuming that the SD may be higher in the current study with the inclusion of outpatients, we would have 90% power detect a 3.9 day or larger mean difference between treatment arms with a SD of 16.

## Additional Details of the Analytic Plan

### *Primary Analyses*

The primary analysis used the Wilcoxon rank sum test, and all subgroup analyses used a two-sided van Elteren test, the stratified version of the Wilcoxon rank sum test.^14^ For each stratum, estimation, and 95% confidence intervals of differences between arms used the Mann-Whitney Parameter, the probability that a subject from the treatment arm has a score greater or equal to that of a subject for the control.^15^ In addition to the primary outcome, the van Elteren test was used to test hypotheses involving the continuous secondary and exploratory outcomes.

### *Per-Protocol Analyses*

In sensitivity analyses, the extent of non-adherence to protocol was described by randomization arm. Cumulative event plots were used to compare non-adherence to the protocol (as a binary event) by randomization arm. If there was a substantial difference observed in non-adherence across treatment arms, we planned *a priori* to adjust for non-adherence in the secondary analyses of the primary endpoint. There was no difference observed in adherence across the treatment arms.

### *Secondary Analyses*

In multivariable adjusted analyses, the FiO_2_/SpO_2_ ratio was normalized for altitude above sea level (atmospheric pressure).

### *Subgroup Analyses*

Exploratory subgroup analyses emphasized estimation and confidence intervals for the purpose of hypothesis generation in future studies as opposed to formal effect modification. In the initial protocol, we planned to perform subgroup analyses of sex, age (categorized by < or ≥ the median value in the study population), race, ethnicity, diagnosis of diabetes at enrollment, BMI (categorized as obese, i.e., BMI ≥30 kg/m^2^ or non-obese i.e., BMI <30 kg/m^2^), inpatient vs. outpatient status at enrollment, FiO_2_/SpO_2_ (categorized by < or ≥ the median value in the study population) at enrollment, and duration of symptoms prior to randomization (<7 vs. ≥ 7 days). Country, baseline disease severity (based on the WHO criteria^16^), and enrollment epoch were recommended as subgroups by our Data Safety Monitoring Board prior to concluding enrollment. Their recommendation acknowledged variation in standard of care among different countries as well as subgroups that were predefined prior to initiation of the study. Fenofibrate formulation was also added as a subgroup analysis due to the use of three different formulations depending on availability within a given country. We also visually explored trends of epoch by country, plotting study month at enrollment vs. summary statistics of the primary outcome by treatment and country.

### *Missing Data*

While missing data is an inevitable problem in longitudinal studies, we made every possible effort to ensure final assessments for all participants, including those opting to discontinue study participation, and only had 18 participants who withdrew from the trial at any point (with only 7 who withdrew prior to collection of any information on the primary endpoint; e.g., the initial post-treatment Borg score assessment). Imputation is not commonly used for rank-based statistical methods such as the van Elteren or Wilcoxon Rank-sum test. If the missingness rate exceeded 5%, we planned to carry out a sensitivity analysis of the primary outcome using linear regression with multiple imputation,^17^ which we did not end up needing to perform. The primary and secondary analyses were complete case analyses.

## Adverse Events

### *Adverse Event Reporting*

Adverse events (AEs) were be captured in our data entry tool (REDCap). The DSMB examined data periodically in order to compare the incidence of adverse events in both arms. Expedited reporting of individual AEs (within 48 hours or discovery by site investigators) was required for severe AEs that were unexpected and felt to be probably or definitely associated with the study intervention. Such expedited reporting of AEs was done from site investigators to the University of Pennsylvania study team, and the study team in turn communicated these to the DSMB.

### *Key definitions*

An AE was defined as any untoward medical occurrence associated with the use of a drug in a study participant whether or not considered drug or biologic related. An AE could therefore be any unfavorable and unintended sign, symptom or disease temporally associated with the use of the pharmaceutical product.

### *Classification of AEs*

A medically-qualified investigator assessed AEs in terms of causal relationship to intervention, seriousness, and expectedness using the following guidelines:

| **Classification of Adverse Events for Causal Relationship to the Study Intervention** | |
| --- | --- |
| Not related or unlikely related | There is not a reasonable causal relationship to the investigational product and the adverse event, or there is a low likelihood that a causal relationship exists. |
| Probably or definitely related | There is reasonable or definite evidence to suggest a causal relationship between the drug and adverse event. |

The events were classified as serious or non-serious adverse events:

*Serious Adverse Events (SAE)* are adverse events, in which the investigator or sponsor believe that any of the following outcomes occur:

- Death
- Life-threatening AE: Places the participant at immediate risk of death at the time of the event as it occurred. It does not include an AE that, had it occurred in a more severe form, might have caused death.
- Persistent or significant incapacity or substantial disruption of the ability to conduct normal life functions.
- Inpatient hospitalization or prolongation of hospitalization.
- Important medical events that may not result in death, be life threatening, or require hospitalization may be considered a serious adverse event when, based upon appropriate medical judgment, they may jeopardize the participant and may require medical or surgical intervention to prevent one of the outcomes listed in this definition above.

*Expectedness:* Expected AEs were those that were previously reported to be associated with COVID-19.

The following AEs are examples of those that were considered expected, disease-related events in patients with COVID-19:

1. Arrhythmias, including ventricular arrhythmias and atrial fibrillation.
2. Acute coronary syndromes with or without coronary stenoses on coronary angiography
3. Myocarditis or worsening cardiac function.
4. Shortness of breath, pneumonia, acute respiratory distress syndrome and respiratory failure.
5. Fever, malaise and myalgia.
6. Worsening cognitive function.
7. Limb ischemia, coagulopathy, thrombosis, embolus.
8. Hypotension or hypertension.
9. Diarrhea, nausea, or vomiting.
10. Anosmia (loss of sense of smell).
11. Leukopenia or leukocytosis, thrombocytopenia.
12. Worsening renal function (resulting in various electrolyte abnormalities).
13. Worsening liver function.
14. Death.

## Protocol Deviations

One participant continued to receive the study medication for 3 days after developing acute kidney injury with an eGFR <30 mL/min/1.73m^2^, which was an indication for early termination of the study medication as per protocol. Two participants were enrolled who unknowingly had exclusion criteria at the time of enrollment (one had a prior history of cholecystectomy and the other had hypothyroidism); both participants were withdrawn from the study as soon as the study team became aware of these elements of the past medical history. One participant did not receive trial medication while hospitalized on one day because the medication could not be located by the nurse on the unit and the study team was not made aware until the following day, when it was readministered. One participant’s 15-day symptom call was not performed within the permitted timeframe due to an oversight of the study team. Six participants in Peru did not have transaminases performed either at baseline or five days after enrollment as required by the study protocol for safety monitoring in that country. Two witnesses of the informed consent entered the incorrect document identification number at the time of consenting; the participants were subsequently reconsented. Three participants initially signed the wrong version of the consent form and were subsequently reconsented using the correct version.

## Study Drug Preparations

The specific preparations utilized in each country were as follows:

| **Country** | **Preparation** |
| --- | --- |
| USA | Tricor (fenofibrate nanoparticle formulation; full dose: 145 mg). The daily dose of Tricor for patients with CKD stage 2 was 96 mg/d and the dose for CKD stage 3 was 48 mg/d. |
| Lebanon | Nanofib (fenofibrate nanoparticle formulation; full dose: 145 mg) |
| Peru | Controlip (micronized fenofibrate; full dose: 160 mg) |
| Mexico | Controlip (micronized fenofibrate; full dose: 160 mg) |
| Colombia | Tripilix (Fenofibric acid; full dose: 135 mg;) |
| Greece | Lipidil (fenofibrate nanoparticle formulation; full dose: 145 mg) |

## Table S1. Components of the global rank score by study arm

| *Variable* |  | *Total (n=701)* | *Placebo (n=350)* | *Fenofibrate (n=351)* |
| --- | --- | --- | --- | --- |
| Death | N (%) | 41 (6%) | 22 (6%) | 19 (5%) |
| Time to death, days | Mean (SD) | 13 (8) | 12 (9) | 13 (6) |
| Invasive mechanical ventilation/ECMO* | N (%) | 46 (7%) | 27 (8%) | 19 (5%) |
| Duration of mechanical ventilation/ECMO, days* | Mean (SD) | 15 (11) | 15 (10) | 16 (11) |
| FiO_2_/SpO_2_^†^ | Mean (SD) | 0.19 (0.49) | 0.19 (0.48) | 0.18 (0.49) |
| Hospitalization^†^ | N (%) | 5 (1%) | 4 (1%) | 1 (<1%) |
| Duration of hospitalization, days^†^ | Mean (SD) | 9 (6) | 10 (7) | 5 (N/A) |
| Borg score during follow-up^†^ | Mean (SD) | 0.6 (1.2) | 0.5 (1.1) | 0.6 (1.3) |
| Pooled COVID-19 symptom score during follow-up^†^ | Mean (SD) | 7 (9) | 7 (9) | 8 (10) |

*Restricted to participants enrolled as inpatients who did not experience the endpoint in the immediately preceding tier, where relevant (e.g., invasive mechanical ventilation/ECMO was restriction to those enrolled as inpatients who did not die)

†Restricted to participants enrolled as outpatients who did not experience the endpoint in the immediately preceding tier, where relevant

*Abbreviations:* COVID-19=coronavirus disease 2019; ECMO=extracorporeal membrane oxygenation; FiO_2_/SpO_2_=fraction of inspired oxygen concentration/percent oxygen saturation; SD=standard deviation

## Table S2. Adverse events by study arm

| *Variable* | *Total (n=701)* | *Placebo (n=350)* | *Fenofibrate (n=351)* |
| --- | --- | --- | --- |
| Serious AEs | 107 (15%) | 61 (17%) | 46 (13%) |
| Death | 41 (6%) | 22 (6%) | 19 (5%) |
| Kidney | 37 (5%) | 22 (6%) | 15 (4%) |
| Hepatic | 48 (7%) | 25 (7%) | 23 (7%) |
| Cardiovascular | 26 (4%) | 12 (3%) | 14 (4%) |
| Respiratory | 76 (11%) | 41 (12%) | 35 (10%) |
| Gastroenterologic | 28 (4%) | 9 (3%) | 19 (5%) |
| Infectious | 55 (8%) | 25 (7%) | 30 (9%) |
| Intensive care unit transfer | 29 (4%) | 16 (5%) | 13 (4%) |
| Delirium | 9 (1%) | 3 (<1%) | 6 (2%) |
| Neurologic | 8 (1%) | 4 (1%) | 4 (1%) |
| Dermatologic | 4 (<1%) | 2 (<1%) | 2 (<1%) |
| Ophthalmologic | 1 (<1%) | 0 (0%) | 1 (<1%) |
| Endocrinologic | 3 (<1%) | 2 (<1%) | 1 (<1%) |
| Hematologic | 22 (3%) | 12 (3%) | 10 (3%) |
| Musculoskeletal | 8 (1%) | 4 (1%) | 4 (1%) |

*Abbreviations: AE=adverse event*

## Figure S1. Kaplan Meier curve of time to hospitalization (among participants enrolled as outpatients)


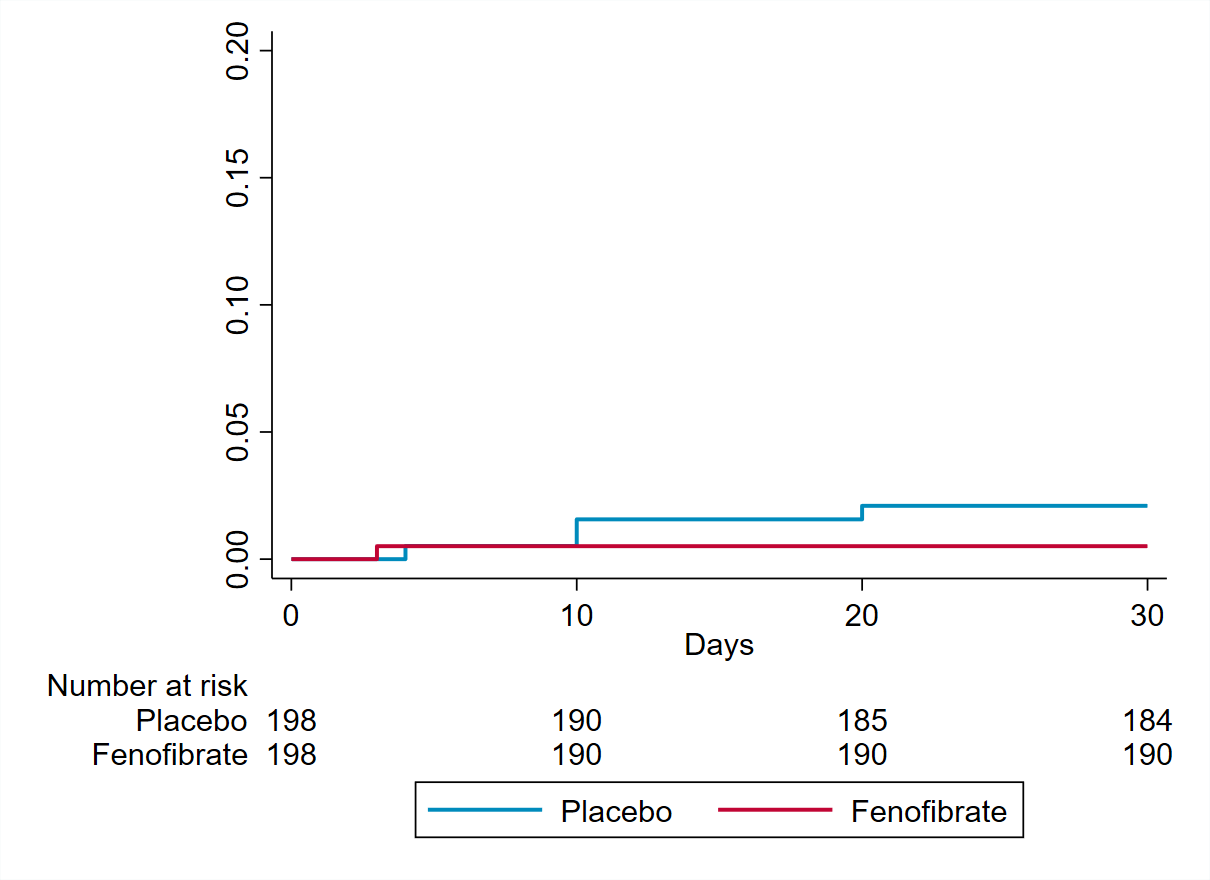


## Figure S2. Cumulative incidence curve of time to discharge (among participants enrolled as inpatients)


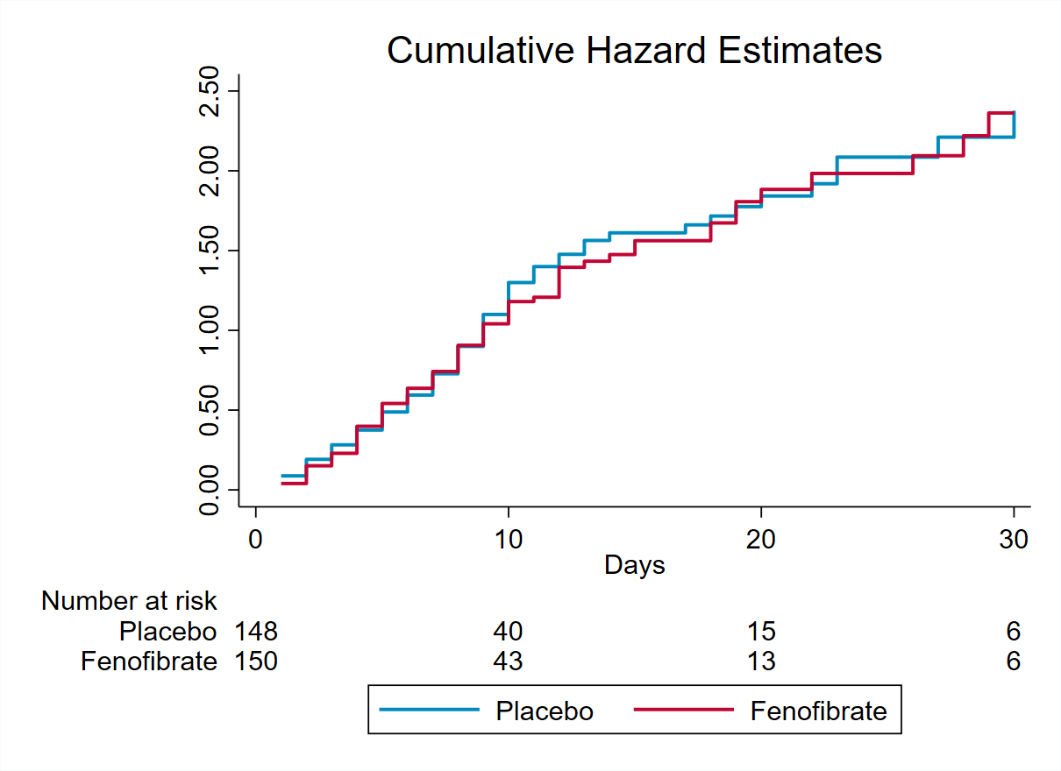


## Figure S3. General workflow of the trial

**
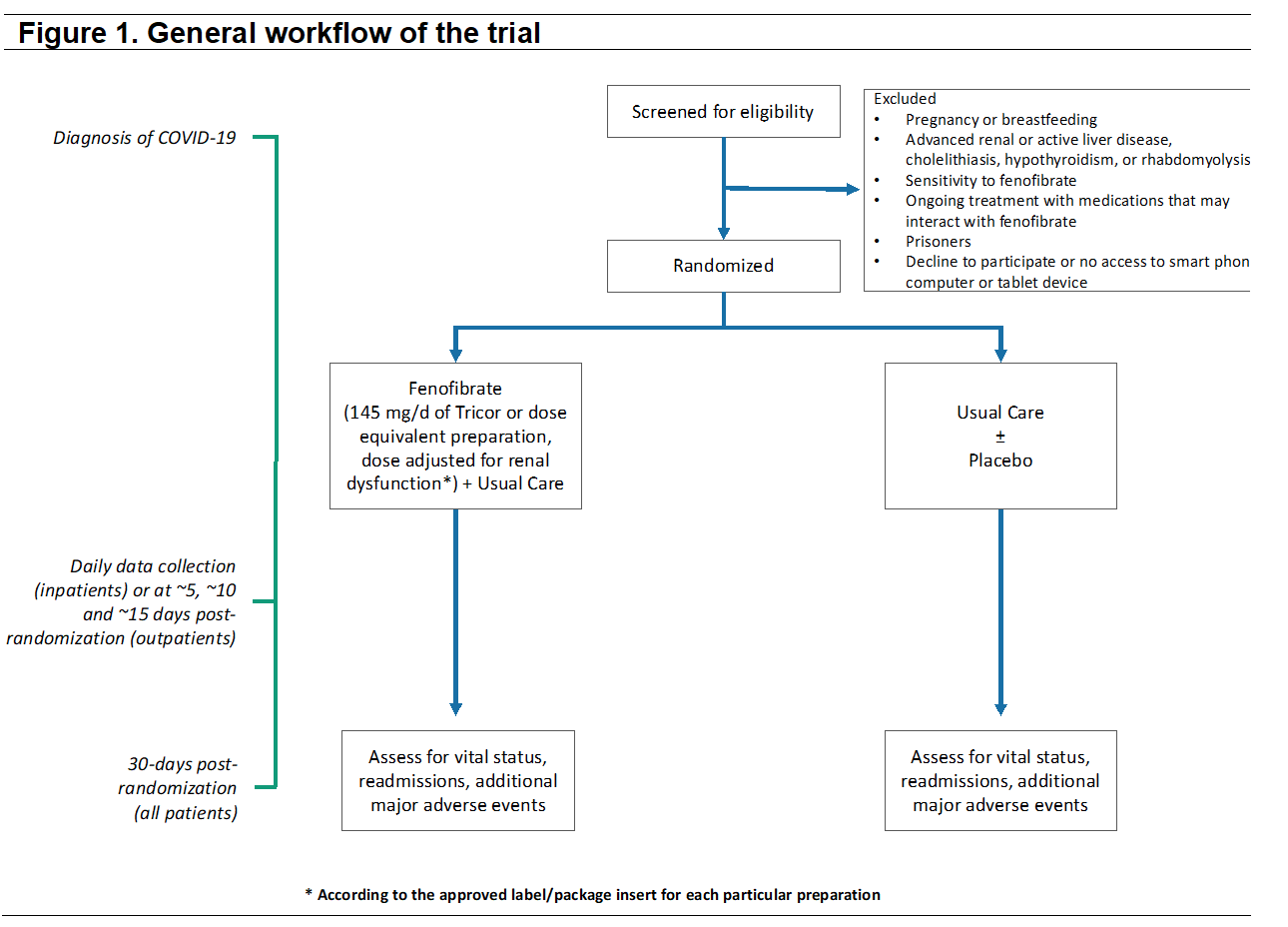
**

## Supplemental References

1. Zhou F, Yu T, Du R, et al. Clinical course and risk factors for mortality of adult inpatients with COVID-19 in Wuhan, China: a retrospective cohort study. *Lancet* 2020; **395**(10229): 1054-62.

2. Wang D, Hu B, Hu C, et al. Clinical Characteristics of 138 Hospitalized Patients With 2019 Novel Coronavirus-Infected Pneumonia in Wuhan, China. *JAMA* 2020.

3. Hozo SP, Djulbegovic B, Hozo I. Estimating the mean and variance from the median, range, and the size of a sample. *BMC Med Res Methodol* 2005; **5**: 13.

4. Julious SA. Tutorial in Biostatistics. Sample sizes for clinical trials with Normal data. *Statistics in medicine* 2004; **23**: 1921-86.

5. O'Brien PC, Fleming TR. A multiple testing procedure for clinical trials. *Biometrics* 1979; **35**(3): 549-56.

6. PASS 16 Power Analysis and Sample Size Software. NCSS, LLC. Kaysville, Utah, USA, ncss.com/software/pass. 2018.

7. Cohen JB, Hanff TC, Corrales-Medina V, et al. Randomized elimination and prolongation of ACE inhibitors and ARBs in coronavirus 2019 (REPLACE COVID) Trial Protocol. *J Clin Hypertens (Greenwich)* 2020; **22**(10): 1780-8.

8. Julious SA. Sample Sizes for Clinical Trials. Boca Raton, FL: Chapman & Hall/CRC; 2010.

9. Chow SC, Shao J, Wang H. Sample Size Calculations in Clinical Research. 2nd Edition ed. New York: Dekker; 2008.

10. Beigel JH, Tomashek KM, Dodd LE, et al. Remdesivir for the Treatment of Covid-19 - Final Report. *The New England journal of medicine* 2020; **383**(19): 1813-26.

11. Whitehead J. Sample size calculations for ordered categorical data. *Statistics in medicine* 1993; **12**(24): 2257-71.

12. Hsieh FY, Lavori PW. Sample-size calculations for the Cox proportional hazards regression model with nonbinary covariates. *Control Clin Trials* 2000; **21**(6): 552-60.

13. Schoenfeld DA. Sample-size formula for the proportional-hazards regression model. *Biometrics* 1983; **39**(2): 499-503.

14. van Elteren PH. On the combination of independent two sample tests of Wilcoxon. *Bull Internat Statist Inst* 1960; **37**: 351-61.

15. Fay MP, Malinovsky Y. Confidence intervals of the Mann-Whitney parameter that are compatible with the Wilcoxon-Mann-Whitney test. *Statistics in medicine* 2018; **37**(27): 3991-4006.

16. WHO R&D Blueprint. Novel Coronavirus. COVID-19 Therapeutic Trial Synopsis Draft. 18 Feb 2020. <https://www.who.int/blueprint/priority-diseases/key-action/COVID-19_Treatment_Trial_Design_Master_Protocol_synopsis_Final_18022020.pdf>. Accessed 28 Apr 2020.

17. Li P, Stuart EA, Allison DB. Multiple Imputation: A Flexible Tool for Handling Missing Data. *JAMA* 2015; **314**(18): 1966-7.
